# Protein interaction networks as metric spaces: a novel perspective on distribution of hubs

**DOI:** 10.1186/1752-0509-8-6

**Published:** 2014-01-18

**Authors:** Emad Fadhal, Junaid Gamieldien, Eric C Mwambene

**Affiliations:** 1 South African National Bioinformatics Institute, SA Medical Research Council Bioinformatics Unit, University of the Western Cape, Bellville 7535, South Africa; 2 Department of Mathematics and Applied Mathematics, University of the Western Cape, P/Bag X17, Bellville, South Africa

**Keywords:** Protein interaction networks, Metric spaces, Core-periphery structure, Topological centrality, Hubs, Essential proteins, Power-law graphs

## Abstract

**Background:**

In the post-genomic era, a central and overarching question in the analysis of protein-protein interaction networks continues to be whether biological characteristics and functions of proteins such as lethality, physiological malfunctions and malignancy are intimately linked to the topological role proteins play in the network as a mathematical structure. One of the key features that have implicitly been presumed is the existence of hubs, highly connected proteins considered to play a crucial role in biological networks. We explore the structure of protein interaction networks of a number of organisms as metric spaces and show that hubs are non randomly positioned and, from a distance point of view, centrally located.

**Results:**

By analysing how the human functional protein interaction network, the human signalling network, *Saccharomyces cerevisiae*, *Arabidopsis thaliana* and *Escherichia coli* protein-protein interaction networks from various databases are distributed as metric spaces, we found that proteins interact radially through a central node, high degree proteins coagulate in the centre of the network, and those far away from the centre have low degree. We further found that the distribution of proteins from the centre is in some hierarchy of importance and has biological significance.

**Conclusions:**

We conclude that structurally, protein interaction networks are mathematical entities that share properties between organisms but not necessarily with other networks that follow power-law. We therefore conclude that (i) if there are hubs defined by degree, they are not distributed randomly; (ii) zones closest to the centre of the network are enriched for critically important proteins and are also functionally very specialised for specific 'house keeping’ functions; (iii) proteins closest to the network centre are functionally less dispensable and may present good targets for therapy development; and (iv) network biology requires its own network theory modelled on actual biological evidence and that simply adopting theories from the social sciences may be misleading.

## Background

In the post-genomic era, an overarching question in the analysis of protein-protein interaction (PPI) networks continues to be whether biological characteristics and functions of proteins such as lethality, physiological malfunctions and malignancy are intimately linked to the topological role proteins play in the network [1,2]. It has been established that protein interaction networks (PINs) have small-world and scale-free properties [3]. Much of the recent efforts in the analysis of protein-protein interaction networks has focused on finding functional dependencies between the so-called hubs and their topological roles in the network [4,5]. What has made the effort the more difficult is that various researchers have defined hubs from various points of view [6]. However, almost universally, it has been assumed that these hubs are proteins having a high degree (number of interactions) and that are randomly placed in the network and have important functional roles [4,7-10]. In a sense, it has been assumed that each hub or a specialised set of hubs somehow controls a sub-network that may constitute a pathway, functional module or a process [11-13]. Because of this, in defining many of the proposed metrics in PINs, the degree of nodes has prominently featured. It is appealing to give substantial import to these metrics because some of them show levels of statistical significance that may not easily be dismissed.

In that respect, for instance, it has been shown that in PINs, proteins of high degree are three times more likely to be lethal than the other proteins [5]. Due to this observation, network theory metrics such as degree centrality, closeness centrality [14], betweenness centrality [15], and cluster coefficients [16], to name a few, have been proposed as ways to identify functionally important proteins in PINs. Whilst these metrics have their relevance, they do not enable the evaluation and analysis of networks as topological entities. Many previous studies on the analysis of complex network used closeness centrality as a way to find the core central proteins and applied it in several areas such as to extract the metabolic core of a network [17], to visualise large scale complex networks in two dimensions [18], to identify drug targets [19,20], to identify essential genes in *Escherichia coli*[21] and to determination of dynamics of the cell-cycle networks [22]. Moreover, they used centrality measures which ultimately give weighting to nodes and do not attempt to identify the exact positions of nodes within the network [23]. Previously considered metrics have, however, implicitly assumed randomness of the distribution of hubs [3,24,25]. Because of the commonality in distribution of networks that follow power-law, some have gone as far as considering PINs in the same manner as social networks [26]. In point of fact, networks of protein interactions have been shown in separate organisms to form, in varying degree of statistical significance, scale-free networks in which the distribution of degrees of nodes is power-law [27,28]. It has thus been assumed that this property captures the essence of the pertinent features of the networks. In this article, it will become evident that paralleling the systems theory that has been developed in social networks to biological networks may not reveal a clearer picture.

The point of departure from these previous studies is that we model PPI graphs as metric spaces, which are well-defined topological spaces with a long history and deep theory [29,30]. This precise strategy provides a powerful way to view PINs in their entirety from a spatial point of view, using distance as the key modeling measure. While we do not attempt to weight proteins, it enables us to pinpoint *exactly* where nodes are located in respect to each other, even in very large PINs with several hundred thousands of proteins and many interactions. By identifying the network centre(s) using a formal method that identifies the protein(s) that have the smallest maximal distance to other proteins in the network, and then categorizing all proteins into zones based on distance from the centre, we are able to find exactly where any protein is located relative to the centre and its corresponding neighbours in the network at large. We show here that modeling and analysing PINs from several sources and organisms in this much more precise manner than can be achieved with centrality metrics reveals deep shared core-periphery topological pattern, and we also present strong evidence of its functional significance.

## Results and discussion

### Multiple protein interaction networks from different organisms all have single topological centres

While the Human Functional Protein Interaction Network (HPFIN), the Human Signalling Network (HSN) and multiple sources of *Saccharomyces cerevisiae*, *Arabidopsis thaliana* and *Escherichia coli* PINs all appear to be power-law distributed as expected (see Additional file 1: Figures S1a, S1b, S1c, S1d and S1e), we modelled each one of the PINs as metric spaces in order to identify each network’s topological centre(s) and to classify remaining proteins into ‘zones’ based on graph theoretic distance from the central protein. Zone 1, for example, refers to proteins which are 1 step from the topological centre, zone 2 is 2 steps away, etc. By modelling the giant component of each PIN in this manner, we found that despite our method’s inevitable ability to identify multiple topological centres if they exist, all PINs analysed had only a single central protein that have key biological functions. The centres of the HFPIN and the HSN, *MAPK14* and *MAPK1* respectively, belong to the same protein family and play similar key roles in signal transduction [31] and the centre of the *Arabidopsis thaliana* PIN is *AT1G78300*, which plays a key role in brassinosteroid mediated signaling [ http://www.ncbi.nlm.nih.gov/pubmed/17681130]. *Saccharomyces cerevisiae* has *SSC1*, involved in stress response [ http://www.molbiolcell.org/content/22/5/541.full] at the centre of its PIN and *Escherichia coli* has *rpsB*, a ribosomal protein S2 that is an essential component of the organisms translation machinery [ http://www.ncbi.nlm.nih.gov/pubmed/23104805].

### PINs analysed as metric spaces form a dense core/sparse periphery structure

The majority of proteins in PINs are located in zones 1–3. For the HFPIN and the HSN respectively, 92% and 95% proteins are in these zones. The same phenomenon is observed in *Saccharomyces cerevisiae*, *Arabidopsis thaliana* and *Escherichia coli*, albeit with varying proportionality (Table 1). When analysing zones as induced sub-networks, we found in all cases that zone 1 is the most highly connected with few quills, if any. Zone connectivity decreases with increasing distance from the centre, with zone 2 having some quills (nodes with degree 1) and zone 3 having many more quills (see the numbers of quills in Table 1). Beyond zone 3, due to very low connectivity, the induced subgraphs disintegrate into many components and in the zones on the fringes there are only quills.

**Table 1:** Degree distribution of PINs with respect to the centre

**Organism**	**Nodes**	**Edges**	**Diameter**	**Centre**	**Zones around centre**									
					**1**	**2**	**3**	**4**	**5**	**6**	**7**	**8**	**9**	
Homo sapiens	9448	181706	13	MAPK14	374	4610	3464	578	104	14	2	1	1	Nodes
					86	32	52	2	2	1	1	2	1	Average degree
					3	1	1	1	1	1	1	2	1	Lowest degree
					531	430	393	14	6	2	2	2	1	Highest degree
					0	173	653	307	56	12	1	0	1	No of quills
HSN	6291	62737	11	MAPK1	431	3527	1929	206	38	4				Nodes
					67	24	7	2	2	3				Average degree
					1	1	1	1	1	1				Lowest degree
					451	362	89	11	9	5				Highest degree
					4	404	757	133	20	2				No of quills
Saccharomyces cerevisiae	5033	22417	10	SSC1	175	1639	2517	565	69	9				Nodes
					34	14	4	1	1	1				Average degree
					1	1	1	1	1	1				Lowest degree
					209	282	109	11	4	1				Highest degree
					1	92	609	327	57	9				No of quills
Arabidopsis thaliana	2953	6783	16	AT1G78300	134	216	799	825	443	130	20	5	4	Nodes
					3	11	6	3	2	1	1	3	2	Average degree
					1	1	1	1	1	1	1	1	1	Lowest degree
					62	117	79	43	26	12	4	6	3	Highest degree
					81	60	180	355	234	80	10	1	1	No of quills
Escherichia coli	2949	12689	12	rpsB	151	1089	976	260	38	6				Nodes
					37	12	3	1	1	1				Average degree
					1	1	1	1	1	1				Lowest degree
					178	127	56	6	3	2				Highest degree
					1	213	349	166	30	5				No of quills

An interesting pattern emerged when we assessed the distribution of the top 5% most highly connected proteins of each network across its zones. Overall, we see that these proteins coagulate in zones closer to the centre of the networks (Tables 2, 3, 4, 5, 6 and 7). When removing a potentially erroneous dense complete subgraph in zone 3 of the HFPIN consisting of 330 zinc finger proteins, the majority of the most highly connected proteins are located in zones 1 and 2. The same is observed for both the HSN and *E. coli*. While *Saccharomyces* and *Arabidopsis* PINs have a similar general pattern, they also have some of these proteins in zone 3, with the latter having an additional 9% in its zones 4 and 5.

**Table 2:** Distribution of top 5% highest degree in human signalling network

**Zone**	**Number of nodes has degree ≥ 95 (5%)**	**Percentage**
Zone 1	106	34.6%
Zone 2	197	64.4%
Zone 3	0	0%
Total	306	100%

**Table 3:** Distribution of top 5% highest degree in HFPIN without complete graph

**Zone**	**Number of nodes has degree ≥ 200 (5%)**	**Percentage**
Zone 1	39	54.9%
Zone 2	32	45.1%
Zone 3	0	0%
Total	71	100%

**Table 4:** Distribution of top 5% highest degree in HFPIN with complete graph

**Zone**	**Number of nodes has degree ≥ 200 (5%)**	**Percentage**
Zone 1	39	8.5%
Zone 2	32	7.6%
Zone 3	380	83.9%
Total	458	100%

**Table 5:** Distribution of top 5% highest degree in Saccharomyces cerevisiae

**Zone**	**Number of nodes has degree ≥ 35 (5%)**	**Percentage**
Zone 1	65	26.5%
Zone 2	152	62%
Zone 3	28	11.4%
Total	245	100%

**Table 6:** Distribution of top 5% highest degree in Escherichia coli

**Zone**	**Number of nodes has degree ≥ 44 (5%)**	**Percentage**
Zone 1	46	37%
Zone 2	77	62%
Zone 3	1	0.8%
Total	124	100%

**Table 7:** Distribution of top 5% highest degree in Arabidopsis thaliana

**Zone**	**Number of nodes has degree ≥ 16 (5%)**	**Percentage**
Zone 1	2	1.5%
Zone 2	46	33.3%
Zone 3	64	44.1%
Zone 4	12	8.5%
Zone 5	5	3.8%
Total	129	100%

**Figure 1:**
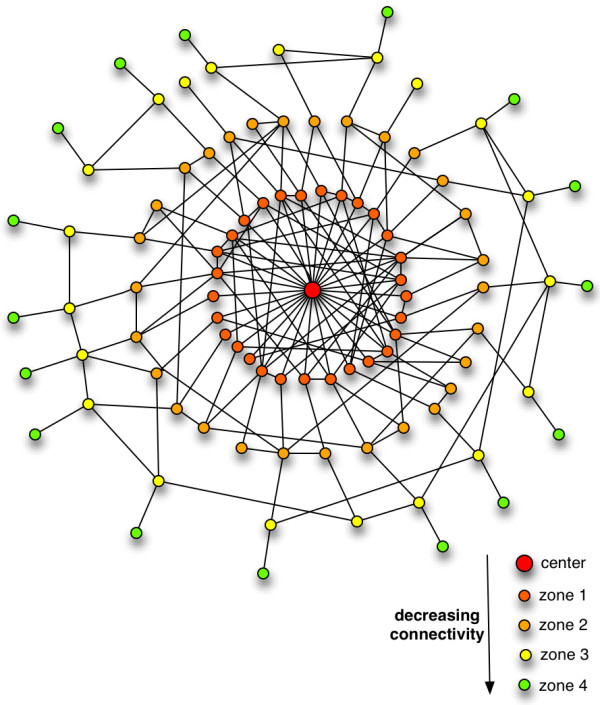
**Model representation of a PINs with respect to distance from the centre.** A graph layout representation of the PINs, which demonstrates a densely connected centre and 'quills’ at the periphery.

It is therefore clear that across the phyla and regardless of network size, PINs form a structure that has a densely connected kernel and a less dense periphery, which terminates in ‘quills’ or ‘spikes’ (Figure 1). Furthermore, topologically central zones are highly connected and have only few proteins of low degree, which suggests a non-random distribution of hub proteins. It has previously been shown that evolutionarily older proteins have higher degree [32] and that loss and gain of protein-protein interaction sites are driven by evolutionary mechanisms [33] and therefore constrained by positive (Darwinian) and negative (purifying) selection. We therefore deem it reasonable to conclude that the core-periphery topologies uncovered by our method are themselves evolved and have functional significance and importance.

There is no phenomenal change (see Additional file 1: Figures S2, S3a, S3b, S3c and S3d) when the analysis is done on different PINs from different database sources. All the results from the analysis of PINs when treated as metric spaces confirm that PINs have a densely connected kernel and becomes less dense towards the periphery, terminating in several ‘spikes’ or ‘quills’.

### Protein network topologies are not consistent with random graphs

**Figure 2:**
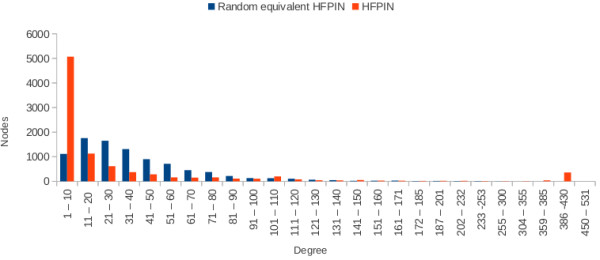
Degree distribution of HFPIN vs equivalent uniform random power-law graph.

**Figure 3:**
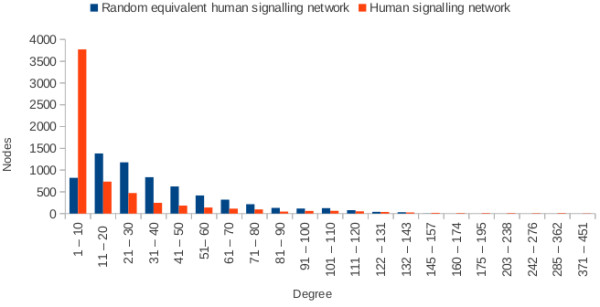
Degree distribution of human signalling network vs equivalent uniform random power-law graph.

The striking similarity across the kingdoms suggests that these network topologies have functional significance and are evolved rather than random, especially since the number of proteins and interactions differ widely. In order to test this hypothesis, we performed a comparison of the biological networks to a large number of computationally-generated uniform random power law graphs [34] with similar properties in terms of numbers of nodes and edges (interactions). In general, while all PINs have a single centre and large diameter, their random power-law equivalents often have multiple centres and significantly smaller diameters. PPI datasets also represented many components with a *single* giant component, while the random graphs consistently had at most 2 components. The number and distribution of quills, nodes of degree 1, is also remarkably different, with PINs having a high number of nodes with low degree in comparison to uniform random power-law graphs (Table 8). This distorts archetypical power-law distribution of nodes. Furthermore, each PIN has a small number of nodes that have remarkably higher degrees than the highest degree nodes of the uniform random power-law graphs (Figures 2 and 3). The significant incongruence of topological patterns between PINs and random graphs add further plausibility to our hypothesis that the biological networks arose through positive selection.

**Table 8:** Human signalling network vs equivalent random human signalling network

**Network**	**Nodes**	**Edges**	**Giant**	**Diameter**	**Zones around centre**						
					**1**	**2**	**3**	**4**	**5**	**6**	
HSN	6291	62737	67	11	431	3527	1929	206	38	4	Nodes
					67	24	7	2	2	3	Average degree
					1	1	1	1	1	1	Lowest degree
					451	362	89	11	9	5	Highest degree
					4	404	757	133	20	2	No of quills
Random equiv. HSN 1	6477	64319	2	8	155	3031	3149	134	5		Nodes
					54	27	11	3	1		Average degree
					7	1	1	1	1		Lowest degree
					150	141	61	10	1		Highest degree
					0	3	32	36	5		No of quills
Random equiv. HSN 2	6270	62360	1	8	151	3037	2973	107	1		Nodes
					56	27	11	2	1		Average degree
					5	1	1	1	1		Lowest degree
					159	136	50	6	1		Highest degree
					0	1	42	32	1		No of quills
Random equiv. HSN 3	6267	62412	1	7	171	3187	2812	96			Nodes
					52	27	11	2			Average degree
					6	1	1	1			Lowest degree
					171	153	58	7			Highest degree
					0	1	39	33			No of quills
Random equiv. HSN 4	6277	62378	2	7	158	3127	2883	106			Nodes
					56	27	11	2			Average degree
					5	1	1	1	1		Lowest degree
					153	143	54	7			Highest degree
					0	2	29	36			No of quills
Random equiv. HSN 5	6282	62378	1	8	154	3093	2914	119	1		Nodes
					55	27	11	3	1		Average degree
					4	2	1	1	1		Lowest degree
					153	158	53	8	1		Highest degree
					0	0	25	34	1		No of quills

### Central zones of human PINs are functionally specialised

**Figure 4:**
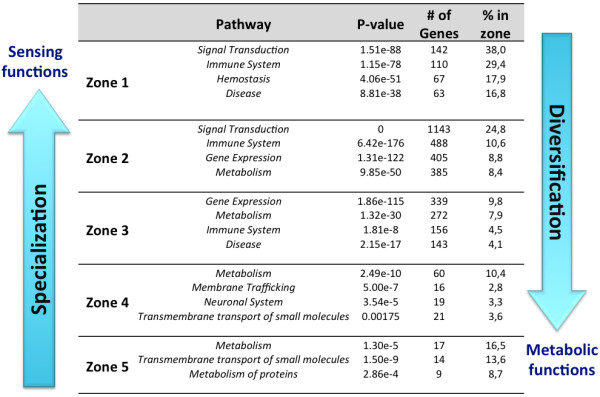
Summary of functional specialization in the central zones of HFPIN.

In order to assess whether the observed topological patterns have potential functional significance, we performed pathway enrichment analysis and observed strong zone-specific functional enrichment in the first four zones of the HFPIN and the HSN. Moreover, those zones also appear to be functionally specialised with most proteins in a zone belonging to the top four enriched pathways, while the outer zones are much more functionally diversified (Table 9, Additional file 1: Table S1). Zone 1 is highly enriched for proteins involved in signal transduction, immune system, hemostasis and disease pathways and appears to constitute a core of highly important interactions required for organismal and cellular sensing and response to adverse environmental, biological and mechanical stresses. Zone 2 is also enriched for proteins involved in signal transduction and immune system pathways and is moderately enriched for gene expression and metabolic pathways, which are the main functional themes in zone 3. Zone 4 has significantly less enrichment than zones closer to the centre, with metabolism and membrane trafficking being the main functional themes for HFPIN and HSN, respectively. Based on pathway enrichment observed in each zone and the high degree of functional specialisation observed in zones closest to the network topological centre, it is likely that the structure of the HFPIN and the HSN (and possibly those of other organisms) may have strong biological significance. We propose that proteins closest to the network centre play critical roles in organismal survival (Figure 4, Additional file 1: Figure S4).

**Table 9:** Summary of functional specialization in the central zones of HFPIN

**Percentage of proteins**				
Enriched pathway	Zone 1	Zone 2	Zone 3	Zone 4
Signal transduction	38%	24.8*%*	-	-
Immune system	29.8*%*	10.6*%*	4.5*%*	-
Hemostasis	17%	5.7*%*	2.7*%*	-
Disease	16.8*%*	8%	4.1*%*	-
Gene expression	8.2*%*	8.8*%*	9.8*%*	-
Metabolism	5.1*%*	8.4*%*	7.6*%*	10.4*%*
Membrane trafficking	-	-	1.2*%*	2.8*%*
Neuronal system	4.8*%*	2.5*%*	2.3*%*	3.3*%*
Transmembrane transport of small molecules	-	1.9*%*	2.4*%*	3.6*%*

### Topologically central proteins may play critical roles in adaptation and survival

In addition to the evidence presented for the human PINs, GO enrichment analysis of central zones of the *Saccharomyces cerevisiae* PIN appears to support our hypothesis that centrally located proteins may be important for organismal fitness, since zone 1 is enriched for functions related to cell cycle, response to stress, reproduction and response to DNA damage and zone 2 for functions related to RNA processing, chromosome organization, ribosome biogenesis and the mitotic cell cycle (Additional file 1: Figure S5). This is further reinforced by our findings that topologically central positions of PINs are highly connected and that hub proteins are located in central zones. Topologically, this is in line with the core and periphery structures described for PPI networks [35]. However, we further propose that the ‘switching’ of specialised functions between zones and the high degree of enrichment for signal transduction proteins in zones 1 and 2 suggests that the human PIN has evolved to optimise the sensing of stimuli at its central zones and to initiate a signal outward to peripheral zones, where transcriptional and subsequent metabolic responses are effected.

## Conclusion

When PINs are formally modelled as metric spaces, it becomes clear that hub proteins are not distributed randomly and that the prevailing view that proteins interact randomly with hubs marshalling low degree proteins in processes and pathways needs a serious reconsideration. We show clearly that PIN structures across the phyla have densely connected kernels and become less dense towards the periphery, terminating in several ‘spikes’ or ‘quills’. We argue that structurally PINs, and possibly other biological networks, are mathematical entities that share properties between organisms but not necessarily with other networks that follow power-law, such as social networks. As such, while applying systems theory developed in social networks to biological networks may have been convenient in and may have shed some light on interactomes, it is not sufficient to identify functional patterns in protein interaction networks, which we have shown to have a much deeper topology when considered as metric spaces. As our over-representation analysis has shown that zones of the human and *Saccharomyces cerevisiae* PINs have functional significance, we argue that interactomics needs its own network theory modelled on actual PPI data rather simply adopting theories from the social sciences.

We thus conclude that our strategy of formally and precisely evaluating PINs as metric spaces, with a focus on zones relative to the centre, may shed light on the key differences between expressed PPI networks in normal and diseased tissues. We propose that centrally located proteins, particularly those involved in sensing functions, may present good therapeutic targets and should be formally evaluated in future studies based on our metric space approach. Our ongoing investigations into the potential applications of the approach detailed in this paper indicates that central zones of several human PPI networks are very strongly enriched for essential proteins and known drug targets, with central zones again displaying high enrichment (data not shown), reinforcing our hypothesis of utility in drug target discovery.

## Methods

### Notation and definitions in graph theory

The PPI networks we consider are modelled by graphs. A *graph* *G* = (*V*,*E*) is a set *V* together with an adjacency relation *E* that is not reflexive and at the same time symmetric. The elements of *V* are called *nodes* (one is a node) and those of *E* are called *edges*. Thus, in PPI networks, proteins are represented by nodes and a pair of proteins forms an edge if they interact; we therefore interchangeably use proteins and nodes in this discussion. In the organisms we consider, to avoid reflexivity, we ignore considerations of proteins interacting with themselves. In any case, in matters of distances, reflexivity plays no role. The *order* of a graph is the number of its nodes and the *size* is the number of interactions. A graph is *complete* if every node is related to the other. If a node *x* is related to a node *y*, we say that *y* is *adjacent* to *x* and write *xy*. The set of nodes that are adjacent to a node *x* is the *neighbourhood* of *x*. The degree of *x* is the number of nodes in its neighbourhood. In the context of PPI networks, the *degree* of a protein is the number of proteins that interact with it. A *subgraph* of a graph *G* is a graph whose node set is a subset of that of *G*, and whose adjacency relation is also a subset of that of *G*. A subgraph *H* of a graph *G* is said to be *induced* if, for any pair of nodes *x* and *y* of *H*, *xy* is an edge of *H* if and only if *xy* is an edge of *G*; that is, *H* inherits all the edges that are in *G*. If a subgraph *H* has only a subset of edges were defined in *G*, then *H* is not induced. Of particular importance in PINs are induced subgraphs that define a process or pathway. A *path* in a graph is a sequence *v*_0_*v*_1_⋯*v*_*k*_ of distinct nodes such that every two consecutive nodes constitute an edge in the graph; its *length* is k. If for every pair of nodes in a graph there is a path joining them, we say that the graph is *connected*; otherwise it is *disconnected*. A *component* is a maximally connected subgraph of the graph and it is *giant* if contains a majority of the entire graph’s nodes. The *distance* between a pair of nodes is the length of a shortest path joining them. A graph together with this distance defines a **metric space**. For a fixed node *v*, the *eccentricity* of *v* is the length of the longest path joining itself to all the other nodes. The longest eccentricity of all nodes is the *diameter* of the graph and nodes with the shortest eccentricity are said to be at the *centre* of the graph. In our context, a *quill* is a subgraph which is on the fringes of the centre and eventually becomes a path, that is, a connected subgraph in which one of the nodes has degree 1.

### PPI data sources

The human PIN we considered is the Human Functional Protein Interaction Network (HFPIN) [36], which has 9448 nodes and 181706 interactions. The human signalling network which has 6291 nodes and 62737 interactions was downloaded from http://www.bri.nrc.ca/wang/. PINs for other organisms were downloaded from various databases: the Database of Interacting Proteins ( http://bioinfo.esalq.usp.br) Version 02/28/2012 [37], bioGRID database ( http://thebiogrid.org/download.php), CCSB interactome database ( http://interactome.dfci.harvard.edu/), antAnc database ( ftp://ftp.ebi.ac.uk/pub/databases/intact/current) Version 2.0, and MINT database ( http://mint.bio.uniroma2.it/mint/download.do). The *Saccharomyces cerevisiae* network consisted of 5033 nodes and 22417 interactions, the *Arabidopsis thaliana* network, 2953 nodes and 6783 interactions and the *Escherichia coli* PPI network consisted of 2949 nodes and 12689 interactions. In order to compare the biological graphs with random graphs, we generated uniform random power-law graphs that are similar in terms of number of nodes and interactions using the Python Webgraph Generator ( http://pywebgraph.sourceforge.net/), which implements the RMAT algorithm [38].

### Evaluation of PPI networks as metric spaces

We considered the Human Functional Protein Interaction Network, the human signalling network, the *Saccharomyces cerevisiae*, *Arabidopsis thaliana*, *Escherichia coli*, *Caenorhabditis elegans* and *Helicobacter pylori* PPI networks as metric spaces by defining the usual graph theoretic distance between nodes of a graph. Using a python wrapper around the C++ BOOST graph library ( http://www.boost.org/), we used the Dijkstra algorithm to compute the shortest distances between *all pairs* of nodes and then identify the node or *all* nodes whose greatest distance to other nodes is/are smallest. This is the network center(s).

From here, nodes were classified according to their distances from the centre and divided into zones based on distance from the topological centre(s). From each distance class, we calculated their degree distributions and also considered their connectivity of the graphs induced for each zone.

### Pathway and function enrichment analysis

In order to determine whether zones of the HFPIN, human signalling network and *Saccharomyces cerevisiae* PIN we considered have biological significance, we divided proteins into subsets based on their distance from the true topological centre. Protein sets representing each zone were then subjected to a pathway over-representation analysis in order to determine whether the zones were specialised for specific functions. The Comparative Toxigenomics Database’s Gene Set Enricher web service ( http://ctdbase.org/tools/enricher.go) and Gene Ontology enrichment ( http://www.geneontology.org/GO.tools) was used to perform the enrichment analysis and a corrected P-value of 0.01 was chosen as a statistical significance cutoff. Lastly, when such enrichment was observed, we calculated the proportion of proteins involved in each enriched pathway as a way to assess whether any zones display functional specialization.

## Competing interests

The authors declare that they have no competing interests.

## Authors’ contributions

EF developed the concept, implemented the algorithms and performed the analysis. ECM proposed the concept of analyzing the PPIs as metric spaces. JG oversaw the functional enrichment analysis and the biological interpretation thereof. ECM and JG designed and supervised the study. All authors have read and approved the final manuscript.

## Supplementary Material

Additional file 1 **Degree distributions of PINs.** In all the PINs, the standard deviation of degree distribution has a remarkably significant variation. However, the HFPIN has a spike as a result of a zinc finger (ZNF) protein family of 330 proteins which constitutes an induced complete graph, where each protein has a degree of 386. Our main strategy was to calculate a number of metrics of networks from their topological centre moving outwards. The correlation coefficient calculated for mean degree and the distance from the centre of the networks is -0.789, -0.814, -0.840, -0.804, -0.865, and -0.876 respectively for HFPIN, human signalling network, *Saccharomyces cerevisiae*, *Escherichia coli*, *Caenorhabditis elegans* and *Helicobacter pylori*. The correlation is therefore strongly negative. In other words, there is a relationship between mean degree and zones. As we move into the centre the values for the mean degree increase. On the other hand, the average degree of nodes in zones in the periphery decrease as one moves away from the centre. Figure S1a Degree distribution of the HFPIN. Figure S1b Degree distribution of *Saccharomyces cerevisiae*. Figure S1c Degree distribution of *Arabidopsis thaliana*. Figure S1d Degree distribution of *Escherichia coli*. Figure S1e Degree distribution of the HSN. Figure S2 Summary of degree distribution of PINs with respect to the centre from different sources. Figure S3a Degree distribution of HFPIN and human signalling network follows power-law distribution. Figure S3b Degree distribution of Saccharomyces cerevisiae from different source. Figure S3c Degree distribution of Arabidopsis thaliana from different source. Figure S3d Degree distribution of Escherichia coli from different source. Figure S4 Summary of functional specialization in the central zones of human signlling network. Figure S5 Summary of functional specialization in the central zones of Saccharomyces cerevisiae. Table S1 Summary of functional specialization in the central zones of human signalling network. Click here for file
