# Supplementary material for: Protein interaction networks as metric spaces: a novel perspective on distribution of hubs
Source: BMC Syst Biol. 2014 Jan 18;8:6. doi: 10.1186/1752-0509-8-6 (PMC3902029; doi:10.1186/1752-0509-8-6)
Supplement: Additional file 1 — Degree distributions of PINs. In all the PINs, the standard deviation of degree distribution has a remarkably significant variation. However, the HFPIN has a spike as a result of a zinc finger (ZNF) protein family of 330 proteins which constitutes an induced complete graph, where each protein has a degree of 386. Our main strategy was to calculate a number of metrics of networks from their topological centre moving outwards. The correlation coefficient calculated for mean degree and the distance from the centre of the networks is -0.789, -0.814, -0.840, -0.804, -0.865, and -0.876 respectively for HFPIN, human signalling network, Saccharomyces cerevisiae, Escherichia coli, Caenorhabditis elegans and Helicobacter pylori. The correlation is therefore strongly negative. In other words, there is a relationship between mean degree and zones. As we move into the centre the values for the mean degree increase. On the other hand, the average degree of nodes in zones in the periphery decrease as one moves away from the centre. Figure S1a Degree distribution of the HFPIN. Figure S1b Degree distribution of Saccharomyces cerevisiae. Figure S1c Degree distribution of Arabidopsis thaliana. Figure S1d Degree distribution of Escherichia coli. Figure S1e Degree distribution of the HSN. Figure S2 Summary of degree distribution of PINs with respect to the centre from different sources. Figure S3a Degree distribution of HFPIN and human signalling network follows power-law distribution. Figure S3b Degree distribution of Saccharomyces cerevisiae from different source. Figure S3c Degree distribution of Arabidopsis thaliana from different source. Figure S3d Degree distribution of Escherichia coli from different source. Figure S4 Summary of functional specialization in the central zones of human signlling network. Figure S5 Summary of functional specialization in the central zones of Saccharomyces cerevisiae. Table S1 Summary of functional specialization in the central zones of human signall [file 1752-0509-8-6-S1.zip › Table S1.pdf]

Table S1 - Summary of functional specialization in the central zones of human signalling network.

Percentage of proteins				
Enriched Pathway	Zone 1	Zone 2	Zone 3	Zone 4
Signal transduction	40.6%	21.1%	9.3%	16%
Immune system	33.6%	16.2%	8.4%	-
MAPK signalling pathway	28.9%	3.5%	-	-
Pathway in cancer	21.4%	5.7%	-	-
Disease	21%	10%	7.2%	9.8%
Hemostasis	16.5%	6.9%	3.3%	-
Cell Cycle	5.3%	7.6%	3.4%	-
Gene expression	7.2%	7.5%	8.7%	-
Metabolism of proteins	-	4.6%	4.6%	-
Membrane trafficking	-	2%	-	4.4%
